# Effects of Service Recovery Expectation and Recovery Justice on Customer Citizenship Behavior in the E-Retailing Context

**DOI:** 10.3389/fpsyg.2021.658153

**Published:** 2021-05-31

**Authors:** Tingting Zhu, Beilei Liu, Mengmeng Song, Jinnan Wu

**Affiliations:** School of Business, Research Institute of Decision and Behavior Science, Anhui University of Technology, Ma’anshan, China

**Keywords:** service recovery expectation, recovery justice, post-recovery satisfaction, customer citizenship behavior, recovery expectation confirmation

## Abstract

Customer citizenship behavior in the online shopping environment is vital to the success of e-retailers. However, it is unclear whether and how service recovery expectation and recovery justice predict customer citizenship behavior in e-retailing settings. Grounded on the expectation confirmation theory and social exchange theory, this study examined the influence of service recovery expectation and recovery justice on customer citizenship behavior with a serial mediation of recovery expectation confirmation and post-recovery satisfaction. A total of 774 samples from e-shoppers with most impressive (*N* = 401) and most recent (*N* = 373) service recovery experience were collected to test the hypotheses using structural equation modeling and bootstrapping procedures. This study reveals that service recovery expectation has a negative impact on recovery expectation confirmation, while recovery justice positively affects recovery expectation confirmation, which is further positively correlated with post-recovery satisfaction and customer citizenship behavior. Moreover, recovery expectation confirmation and post-recovery satisfaction play a serial mediation in the relationship between service recovery expectation and recovery justice, and customer citizenship behavior. Our study contributes to the growing body of customer citizenship behavior literature by offering an alternative perspective (i.e., service recovery) to understand what encourage or impede customer citizenship behavior, and expands service recovery literature by combining service recovery expectation and recovery justice into a framework and revealing the expectation–confirmation mechanism through which they influence post-recovery satisfaction in online shopping setting.

## Introduction

With the rapid development of digital technology, online shopping trends in China are expected to grow rapidly (Xu et al., 2020). Compared with physical stores, e-shops rely more on customer citizenship behavior, which refers to a customer’s self-willingness to take part in unsolicited, helpful, and constructive behaviors toward other customers and the company, to obtain and maintain a competitive advantage (Gong and Yi, 2019; Liu and Lin, 2020). Because e-retailers who face too many imitators and competitors in mature homogenous markets are inclined to rely more on the help and recommendations of e-shoppers (Yi and Kim, 2017; Burnham et al., 2020). Different from offline shopping, e-shoppers are lack of real-time face-to-face interaction with salespersons, and are not able to touch, smell, taste or try on tangible goods before making a purchase (Wu et al., 2020). In addition, uncertainties in logistics distribution and the shortcoming of information leaking are on the rise. So compared with off-line retailing, more service failures may occur in the service delivery process. Further, it is difficult for e-retailers to detect service failures in the online shopping environment (Harris et al., 2006). Once service failure occurs and cannot be effectively recovered, the negative word of mouth will be spread like a virus, which will bring huge losses to e-retailers. Also, the lack of high switching costs enables e-shoppers to switch to a different e-shop with just a single click of the computer mouse. In a word, service failure is more inevitable and influential on consumer behavior in the e-retailing context, and customers’ “initiative” is more valuable to e-retailers than ever before (Anaza and Zhao, 2013). Therefore, encouraging customer citizenship behavior from online service recovery is an important issue that requires urgent attention.

However, research relating to this issue has rarely been conducted, which leads to a need for more work on the customer citizenship behavior and its antecedents in the service recovery setting. Extant studies have examined the antecedents of customer citizenship behaviors, such as customer characteristics (e.g., customer satisfaction, customer commitment, fairness, trust, self-sacrifice, awareness of public self-image, customer expertise, self-efficacy, social capital, positive affect, empathic concern, other-oriented empathy, helpfulness, proactive personality) (Bettencourt, 1997; Groth, 2005; Yi and Gong, 2006; Yi and Gong, 2008; Chen et al., 2010; Di et al., 2010; Anaza, 2014; Curth et al., 2014; Alves et al., 2016; Dang and Arndt, 2017; Choi and Hwang, 2019), other-customer characteristics (e.g., support from other customers, customer-to-customer interaction quality, other-customer citizenship behavior, positive customer-to customer interaction) (Rosenbaum and Massiah, 2007; Yi et al., 2013; Verleye et al., 2014; Kim and Choi, 2016; Jung and Seock, 2017), service characteristics (e.g., service quality, crowding, service scripts, brand experience, brand relationship quality, brand attachment, brand community identification) (Nguyen et al., 2014; Verleye et al., 2014; Cheng et al., 2016; Xie et al., 2017; Wei et al., 2019; Mandl and Hogreve, 2020), employee characteristics (e.g., employee emotional intelligence, employee commitment, employee credibility, employee benevolence, employee loyalty, organizational citizenship behavior) (Yi and Gong, 2008; Bove et al., 2009; Chan et al., 2017; Delpechitre et al., 2018), and organizational characteristics (e.g., organizational legitimacy, organizational support, organizational socialization, organizational support, organizational socialization, organizational identification, organizational reputation) (Bettencourt, 1997; Ahearne et al., 2005; Bartikowski and Walsh, 2011; Verleye et al., 2014; Chen et al., 2019; Kim et al., 2020). However, customer citizenship behavior is underexplored within the context of service recovery to date. The role of service recovery expectation and recovery justice in predicting customer citizenship behavior has not yet been verified. This study uses the logic of expectation confirmation theory (ECT) and social exchange theory (SET) to examine consumers’ behavioral responses to service recovery. Specifically, we examine whether and how service recovery expectation and recovery justice contribute to customer citizenship behavior with serial mediations of recovery expectation confirmation and post-recovery satisfaction.

Although numerous studies have documented the significant direct effect of recovery justice (interactional, procedural, distributive) on improving post-recovery satisfaction in the online/offline context (Cheung and To, 2016; Jung and Seock, 2017; Balaji et al., 2018; Cantor and Li, 2019), the psychological mechanisms that account for the effect is still unclear. Hence, more work is needed to examine how perceived justice of service recovery affects post-recovery satisfaction. Grounded on ECT, this study intends to extend the existing service recovery literature by analyzing the direct effect of recovery justice on post-recovery satisfaction, along with its indirect effect, via expectation confirmation. The direct and indirect effects of recovery justice on post-recovery satisfaction are further examined in an effort to gain in-depth insights into the customer’s evaluation process with service recovery.

Furthermore, although prior studies have investigated the influence of expectation and performance on satisfaction with the mediation of expectation confirmation in the context of offline service recovery (Smith et al., 1999; Andreaseen, 2000; McCollough et al., 2000), little empirical research has been performed to investigate the joint effects of recovery expectation and recovery justice on recovery confirmation and post-recovery satisfaction in online recovery. On the basis of ECT, this study advance our understanding of service recovery literature by exploring how post-recovery satisfaction forms through a confirmation process involving both recovery expectation and recovery justice in online shopping setting.

Specifically, we established a theoretical framework that integrates the ECT with SET to examine how to encourage customer citizenship behavior through online service recovery. From the perspective of ECT, satisfaction is a function of confirmation of expectation and performance (Susarla et al., 2003), which determines customer behavior (Oliver, 1980). From the perspective of SET, if customers are satisfied with a service provider, they may consider the firm as living up to the end of their contractual bargain of providing extra service, causing customers to reciprocate the favor by participating in voluntary unsolicited exceptional role behavior in future transactions (Groth, 2005). This research thus extends customer citizenship behavior literature by integrating the logic of ECT and SET to explain and predict the reaction of e-shoppers to service recovery.

This study contributes to customer citizenship behavior and service recovery literature in three ways. First, we extended the customer citizenship behavior to service recovery context by exploring how recovery expectation and recovery justice affect customer citizenship behavior. Second, we shed light on recovery justice – post-recovery satisfaction mechanisms by investigating the influence of recovery justice on recovery satisfaction through the mediating role of recovery expectation confirmation. Finally, we revealed the joint impacts of recovery expectation and recovery justice on recovery expectation confirmation and post-recovery satisfaction in online shopping setting. Thus, we have developed a more comprehensive framework than previous studies conducted in physical shopping context to understand how to encourage customer citizenship behavior from online service recovery.

The structure of this paper is as follows. First, the theoretical background is introduced, and related literature is reviewed systematically. Next, the hypotheses and research model are put forward. The conceptual model integrates the relationship among the critical variables, especially service recovery expectation, recovery justice and customer citizenship behavior. Third, the research methodology is presented, followed by the results of data analysis. We conclude with a discussion of research findings, the implications for theory and practice, the analysis of limitations, and the suggestions for future research.

## Theoretical Background and Hypotheses Development

### Theoretical Background

#### Customer Citizenship Behavior

Based on Groth’s (2005) definition of customer citizenship behavior, e-shoppers’ customer citizenship behavior refers to the voluntary and discretionary behaviors expressed by e-shoppers in the virtual network environment to promote the delivery, purchase, and consumption of products or services, while in the meantime help the business succeed (Groth, 2005). The extra role that e-shoppers play in the process of service delivery helps e-retailers figure out knowledge-based solutions according to the information offered by customers, which can bring big rewards to e-shops (Anaza and Zhao, 2013).

Due to the important role of customer citizenship behavior in establishing competitive advantages, encouraging customer citizenship behavior has always been a hot issue and has received more and more attention. Scholars have studied the impact of customer characteristics, other-customer characteristics, service characteristics, employee characteristics, and organizational characteristics on customer citizenship behavior. A number of studies pointed out that customer satisfaction and customer citizenship behavior are positively correlated (Bettencourt, 1997; Groth, 2005; Chen et al., 2010). This law can also be applied to commitment, trust, fairness, and loyalty (Yi and Gong, 2006, 2008; Bove et al., 2009; Di et al., 2010; Bartikowski and Walsh, 2011; Curth et al., 2014). Some scholars stressed the importance of customers’ personal traits (e.g., customer expertise, customer emotion, and customer personality) as antecedents of customer citizenship behavior (Yi and Gong, 2008; Anaza, 2014; Alves et al., 2016). Moreover, other customer characteristics have also been found to be very important. Such as, support from other customers (Rosenbaum and Massiah, 2007; Verleye et al., 2014), customer-to-customer interaction quality (Kim and Choi, 2016; Jung and Seock, 2017) and other customer citizenship behavior (Yi et al., 2013) are closely related to customer citizenship behavior. In addition, service characteristics (e.g., service quality, service scripts) are known to increase customer citizenship behavior (Nguyen et al., 2014; Verleye et al., 2014). Brand experience, brand relationship quality, brand community identification and brand attachment are positively associated with customer citizenship behavior (Cheng et al., 2016; Xie et al., 2017; Mandl and Hogreve, 2020). Besides, employee characteristics (e.g., employee emotional intelligence, employee commitment, employee credibility, employee benevolence, employee loyalty) have great influences on customer citizenship behavior (Bove et al., 2009; Delpechitre et al., 2018). Organizational citizenship behavior is proven to be closely linked to customer citizenship behavior (Yi and Gong, 2008; Chan et al., 2017). Further, organizational characteristics, such as, organizational legitimacy (Chen et al., 2019), organizational support (Bettencourt, 1997; Verleye et al., 2014), organizational socialization (Guo et al., 2013), organizational identification (Ahearne et al., 2005), organizational reputation (Bartikowski and Walsh, 2011), corporate social responsibility (Kim et al., 2020), have been confirmed to have effects on customer citizenship behavior. However, little attention has been paid to the influence of customers’ expectation and justice perception on satisfaction and customer citizenship behavior in the service recovery context. The present study seeks to narrow the gap in the customer citizenship behavior research by integrating the ECT with SET to examine how to encourage customer citizenship behavior through service recovery from the perspective of recovery expectation and recovery justice.

#### Service Failure and Recovery

Service failure was defined as a mistake or problem that consumers experience while shopping or communicating with firms, which results in customer dissatisfaction as well as causing potential damage to customer relationships and loss of revenue (Maxham, 2001). Therefore, service recovery measures are used by service providers to recover customer trust damaged by service failures (Weun et al., 2004). Effective service recoveries help to restore the loss of customer satisfaction, promote customer loyalty, and keep a long-term relationship with customers (McCollough et al., 2000; Kuo and Wu, 2012). There are more causes of service failures in the e-retailing context. For example, late delivery, improper packaging, payment security concerns, and personal information leaking (Holloway and Beatty, 2003; Forbes et al., 2005). Also due to more interactive communication, consumers in the e-retailing setting are more informed, knowledgeable and demanding than in physical stores (Miller et al., 2000; Wind and Rangaswamy, 2001). E-retailers are more likely to dissatisfy customers in the e-retailing context, and consumers easily switch e-retailers by several clicks (Shankar et al., 2003). Therefore, it is critical for scholars and practitioners to better understand service recovery in the e-retailing context.

Perceived justice is considered to be a critical factor in the customers’ evaluation of service recovery. Past studies indicated that customers’ perceived justice could directly produce satisfaction during service recovery (Karande et al., 2007; Cheung and To, 2016; Balaji et al., 2018; Chao and Cheng, 2019). Researchers verified significant direct relationship between the three recovery justice dimensions (distributive justice, procedural justice, interactional justice) and customer’s post-recovery satisfaction (Wen and Chi, 2013; Gohary et al., 2016; Jung and Seock, 2017; Cantor and Li, 2019). However, the mechanism through which recovery justice influences post-recovery satisfaction is unexplored. In addition, although prior studies have looked at the effects of recovery expectation and recovery performance on recovery satisfaction with the mediation of expectation confirmation in the offline line environment (Andreaseen, 2000; McCollough et al., 2000), there has been little discussion about the complex interrelationships among expectation, justice, confirmation and satisfaction in the online service recovery context. The current study intends to bridge the gap in the existing literature by testing the mediating role of recovery expectation confirmation in the recovery expectation-post-recovery satisfaction link and recovery justice-post-recovery satisfaction in online shopping setting.

#### Social Exchange Theory

Social exchange theory has been a primary research framework adopted to understand organizational citizenship behavior. The SET is based on the principle that people build and maintain relationships with others because they believe that both parties can benefit from cooperation (Blau, 1964). People believe that the principle of reciprocity has always existed (Homans, 1958), and feel that when they benefit from the actions of others, it is their responsibility to reciprocate (Gouldner, 1960). Research indicates that social exchange between service providers and customers can improve the perceived satisfaction of service encounters (Anaza and Zhao, 2013; Jung and Seock, 2017). Based on the SET, we investigated how e-retailers’ past service recovery experience affects e-shoppers’ behavioral intention, which ultimately affect customer citizenship behavior. More specifically, we assume that in the context of e-retailing, when e-shoppers receive effective service recovery from e-retailers, they will be grateful for the benefits and try to reward e-retailers through positive emotional and cognitive responses and participation in customer citizenship behavior.

#### Expectation Confirmation Theory

Expectation confirmation theory, also known as Expectation–Disconfirmation Theory (EDT), is commonly used in marketing literature to understand consumer satisfaction and post-purchasing behavior of customer (Malik and Rao, 2019). There are five major constructs in the ECT: prior expectation, perceived performance, expectation confirmation, satisfaction, and consumer behavioral intention. ECT suggests that consumers develop a prior expectation for products or services before consumption. After consuming a product or service, they perceive the actual performance of the product or service. Consumers then compare their perceived performance with prior expectation and determine the extent to which his or her prior expectation is confirmed, which in turn determines the level of his or her satisfaction (Kima and Baker, 2020). A satisfied consumer shows a positive behavioral intention, while a dissatisfied consumer spreads the negative word of mouth and turns to other firms (Hossain and Quaddus, 2012). In this study, we used the ECT to understand customer service recovery expectation (prior expectation) and perceptions of justice (perceived performance) in the recovery process (Oliver and Bearden, 1985; Oliver and Swan, 1989). We examined whether a customer’s post-recovery satisfaction and customer citizenship behavior will differ according to whether the recovery efforts matched or did not match his/her prior expectation. In other words, we proposed that customers initially form expectations for service recovery in the event of service failure. After experiencing service recovery, customers perceive the actual recovery performance when the service providers make recovery efforts. When consumers believe that recovery performance meets or exceeds recovery expectation, they experience positive recovery expectation confirmation and their post-recovery satisfaction increases. However, when the recovery performance lags behind the recovery expectation, consumers will undergo negative recovery expectation dis-confirmation, and post-recovery satisfaction diminishes, which determines consumer behavior (Schoefer and Ennew, 2005).

**FIGURE 1:**
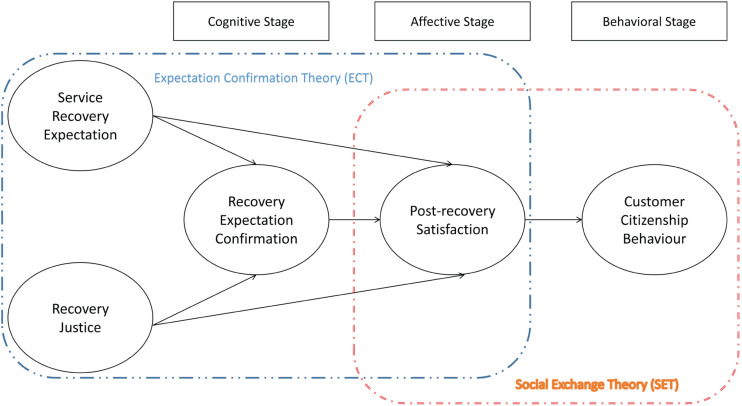
Research model for understanding how service recovery expectation and justice link to customer citizenship behavior.

By integrating SET into ECT, Figure 1 describes the concept model and theoretic hypotheses. The research model describes a sequential framework, starting from an e-shopper’s service recovery expectation and perception of recovery justice, and finally deriving customer citizenship behavior. This study integrates cognitive and affective components with the behavioral component in the conceptual model. Recovery expectation confirmation can be considered as a cognitive component. Post-recovery satisfaction can be viewed as an affective consequence of recovery expectation-recovery justice gap, which refers to a pleasant emotional state which results from the fulfillment of expectations after service recovery experience. Recovery expectation confirmation is assumed to affect post-recovery satisfaction. This study regards customer citizenship behavior as the behavioral outcome of both cognitive and affective components. Post-recovery satisfaction is expected to have a significant impact on the customer citizenship behavior of e-shoppers.

### Hypotheses Development

#### Post-recovery Satisfaction and Customer Citizenship Behavior

Based on the SET, the studies have concluded that there is a strong correlation between customer satisfaction and customer citizenship behavior (Yi and Gong, 2006). Bettencourt (1997) found that customer satisfaction is positively related to customer citizenship behavior. When customers feel obligated to repay the favor of the organization, their reciprocity takes the form of customer citizenship behavior. For example, customers who are satisfied with the firm are inclined to engage in customer citizenship behaviors to return the favor (Groth, 2005). This conclusion may also apply to service recovery situations. When consumers are satisfied with service recovery that exceeds their expectations, they are more likely to engage in reciprocal behavior that may benefit the service provider. Moreover, dissatisfied customers who received effective service recovery are inclined to show higher positive behavioral intentions than those satisfied with the first service encounter. Therefore, appropriate service recovery could improve customer satisfaction and ultimately enhance customer citizenship behavior. In this regard, the following hypotheses were proposed:

H1: Post-recovery satisfaction has a positive effect on customer citizenship behavior.

#### Recovery Expectation Confirmation and Post-recovery Satisfaction

The ECT posits that consumers generate expectations before service recovery based upon experience when they encounter service failure. After service recovery, the process of comparison, in other words, recovery expectation confirmation, leads to (1) positive confirmed status where the perceived recovery performance meets or exceeds the prior expectation and (2) negative dis-confirmed status where the perceived recovery performance is lower than the prior expectation (Oghuma et al., 2016). Several empirical studies explicitly confirmed that individuals are satisfied when outcomes meet or exceed the initial expectations and dissatisfied in the case of negative dis-confirmation (Fu et al., 2018; Nam et al., 2020). Bhattacherjee (2001) stressed that users’ extent of confirmation is positively associated with their satisfaction in the online banking context. Similarly, Lee and Kwon (2011) found that the extent to which users experience confirmation has a positive effect on their satisfaction with a web-based service. While examining service recovery context, McCollough et al. (2000) pointed out that if there is a gap between recovery expectation and recovery performance, the expectation discrepancy will be generated, which will affect post-recovery satisfaction. In line with Boshoff (1997); Andreaseen (2000) considered that post-recovery satisfaction is related to recovery expectation confirmation. Thus, recovery expectation confirmation is positively correlated with post-recovery satisfaction. Therefore, we hypothesize:

H2: Recovery expectation confirmation has a positive effect on post-recovery satisfaction.

#### Effects of Service Recovery Expectation and Recovery Justice on Post-recovery Satisfaction

Expectation has always been regarded as an important source of influencing customers’ judgment and evaluation of a firm and its products or services after a service recovery (Bhattacherjee, 2001; Fu et al., 2018). Oliver (1981) considered expectation as an antecedent of satisfaction. Some studies have found that the higher the customer’s expectations, the lower the satisfaction (Anderson and Sullivan, 1993; Parasuraman et al., 1998). Expectation, as an ex-ante construct, offers a basis for service recovery evaluation. Andreaseen (2000) found that customers’ high level of recovery expectation is possible to reduce the level of post-recovery satisfaction. Hess et al. (2003) also believed that customers with lower service expectations would be more satisfied with recovery efforts. Therefore, we proposed the following hypothesis:

H3: Service recovery expectation has a negative effect on post-recovery satisfaction.

The impact of perceived justice on satisfaction has been examined in the service recovery context. Previous studies have proved that higher perceived levels of justice are positively associated with higher post-recovery satisfaction (Cantor and Li, 2019). Because the level of customer satisfaction and behavioral intentions depend on whether customers feel they receive fair treatment, in other words, whether customers feel justice was implemented (McColl-Kennedy and Sparks, 2003). In line with Cheung and To (2016); Balaji et al. (2018) also pointed out that perceived justice accounts for the largest proportion of the explained variance in recovery satisfaction. Chao and Cheng (2019) showed that customer’s post-recovery satisfaction increases as recovery justice rises, and similar findings have been found in the restaurant industry (Mattila and Patterson, 2004), airline industry (Wen and Chi, 2013), and cell-phone industry (Del Río-Lanza et al., 2009). Further, in the context of e-retailing industry, Gohary et al. (2016) and Jung and Seock (2017) indicated that justice dimensions could win post-recovery satisfaction, because considering justice dimensions from customers’ perspective allow firms to more thoroughly understand customers and improve customers’ post-recovery satisfaction. Based on these arguments, the following hypothesis is proposed:

H4: Recovery justice has a positive effect on post-recovery satisfaction.

#### Effects of Service Recovery Expectation and Recovery Justice on Recovery Expectation Confirmation

Service recovery expectation is considered as a baseline for comparison with the perceived performance of service recovery. It means customer’s prediction of service recovery that may occur in the future, which affects customer’s perception level of service recovery performance (Jomnonkwao et al., 2015). Recovery expectation confirmation depends on the individual’s assessment of perceived service recovery performance and pre-recovery expectations of service recovery (Oliver, 1980). In this regard, higher expectations are more liable to result in the negative dis-confirmed status, and the converse is also true (Fu et al., 2018). McCollough et al. (2000) also noted that the higher (lower) recovery expectations, the less (more) positive recovery confirmation. Service recovery expectation and recovery expectation confirmation are negatively correlated (Yim et al., 2003). Hence, we hypothesize:

H5: Service recovery expectation has a negative effect on recovery expectation confirmation.

In accordance with the ECT, after consuming a given product or service, consumers would develop a perception known as perceived performance. In the event of service failure, consumers usually receive redress as a recovery (Oliver, 1980). As a result, the perception of justice in the process of receiving redress from the service provider becomes the perceived performance of consumers in service recovery (Adams, 1965). According to McCollough et al. (2000), the greater (lower) recovery performance, the more (less) positive recovery confirmation. Recovery justice works as the standard of comparison for recovery expectation confirmation; the higher recovery justice is more liable to increase the positive recovery expectation confirmation (Schoefer and Ennew, 2005). We proposed the following hypothesis:

H6: Recovery justice has a positive effect on recovery expectation confirmation.

#### Serial Mediation Effects of Recovery Expectation Confirmation and Post-recovery Satisfaction

The mediating role of recovery expectation confirmation has been verified in previous research. For instance, Oh (1999) indicated that expectation confirmation completely mediates perceived service quality toward satisfaction. Post-recovery satisfaction is also considered as a mediator in previous research. Westbrook (1987) considered satisfaction as the “central mediator of post-purchase behavior, linking prior product beliefs to post-purchase cognitive structure, consumer communications, and repurchase behavior.” Tax et al. (1998) pointed out that post-recovery satisfaction can mediate the impact of perceived justice on post-complaint evaluations. Wirtz and Mattila (2004) stated that post-recovery satisfaction is the intermediate variable between service recovery characteristics and behavioral intention. Sui et al. (2013) believed that post-recovery satisfaction works as a mediator between justice perception and behavioral intention. Based on the ECT and SET, this study considers that consumers first make predictions about the specific service recovery measure that may be taken in the future in the case of service failure. After experiencing service recovery, consumers develop a perception of justice of service recovery based on his or her experience. Consumers then make a comparison between recovery justice and recovery expectation to determine the extent to which recovery expectation is confirmed, thereby affecting post-recovery satisfaction. Satisfied consumers show positive behavioral intentions, while dissatisfied customers develop negative behavioral intentions. In this regard, the following hypotheses are posited:

H7: Service recovery expectation is negatively associated with customer citizenship behavior via the serial mediation of recovery expectation confirmation and post-recovery satisfaction.

H8: Recovery justice is positively associated with customer citizenship behavior via the serial mediation of recovery expectation confirmation and post-recovery satisfaction.

## Materials and Methods

### Sample and Procedure

In this study, we collected self-reported survey answers using a questionnaire distributed by Wenjuanwang^1^, one of China’s largest online survey website. In contrast with the offline survey, online survey website provides greater anonymity, diversity and recordability, and the collection of sensitive information is more authentic (Stewart and Bing, 2009).

Questionnaires were randomly distributed to e-shoppers with service recovery experience through the Wenjuanwang platform. Regardless of whether the respondent uses a computer or a mobile phone, they can fill out the questionnaire by scanning the quick response code or using a hyperlink. To prevent the same respondent from repeatedly answering the questionnaire, only one questionnaire is allowed for each IP address. Demographic information of each sample was collected and considered as control variables, which may influence customer citizenship behavior, including gender, age, education level, average monthly income, occupation, e-shopping experience, and e-shopping frequency (Kim et al., 2010; Gong and Yi, 2019). To reduce the resistance of the respondents, sensitive demographic questions were placed at the end of the questionnaire, which can increase the response rate (Teclaw and Osatuke, 2012). The rest of the questionnaire contained several multi-item scales that measure the variables of service recovery expectation, perceived justice in service recovery, recovery expectation confirmation, post-recovery satisfaction and customer citizenship behavior in the e-retailing context, which were discussed further.

Before answering all survey questions, respondents were required to refer to the most impressive service failure/recovery experience that they had experienced during online shopping. In this study, the questionnaire was randomly distributed to 600 respondents by the Wenjuanwang platform, and 420 questionnaires were collected. Among them, 19 questionnaires with unfilled or suspected untrue answers were excluded, and 401 valid questionnaires were retained for further analysis, representing a 66.8 percent valid response rate. Table 1 shows the demographics and e-shopping behavior of the respondents. More than half of the respondents were female (61.8%), while about 38.2% were male. Respondents aged under 25 make up 57.6% of the total 234 (58.4%) of the respondents’ average monthly income is 2,501–5,000 RMB, 85 (21.2%) of them earn 5,001–10,000 RMB, 42 (10.5%) of them earn less than 2,500 RMB, 23 (5.7%) of them earn 10,001–15,000 RMB, while 17 (4.2%) of them earn more than 15,001 RMB. The three occupations with the highest percentages are students (32.4%), professionals (16%) and white-collar (12.5%). Nearly all respondents reported having an undergraduate (50.1%) or postgraduate degree (38.9%). In terms of e-shopping experience, most respondents have 4–6 years of experience (44.9%). Regarding e-shopping frequency, many respondents make e-shopping 1–3 times a month (42.6%).

**TABLE 1:** Respondent profile (*N* = 401).

Variable	Category	Frequency	Percentage (%)
Gender	Male	153	38.2
	Female	248	61.8
Age	18–24	231	57.6
	25–30	62	15.5
	31–40	42	10.5
	41–50	53	13.2
	≥51	13	3.2
Education	Senior high school or below	11	2.7
	College degree	33	8.2
	Bachelor’s degree or equivalent	201	50.1
	Master’s degree or higher	156	38.9
Occupation	Student	130	32.4
	Government officials/public servant	38	9.5
	Management/administrative staff	42	10.5
	White collar	50	12.5
	Professionals	64	16.0
	Worker	17	4.2
	Service staff	17	4.2
	Self-employed	15	3.7
	Freelancer	18	4.5
	Others	10	2.5
E-shopping experience	1–2 years	16	4.0
	2–3 years	45	11.2
	3–4 years	45	11.2
	4–6 years	180	44.9
	> 6 years	115	28.7
E-shopping frequency per month	1–3 times	171	42.6
	4–7 times	131	32.7
	8–10 times	39	9.7
	> 11 times	60	15.0
Average monthly income	≤¥2500	42	10.5
	¥2501–5,000	234	58.4
	¥5,001–10,000	85	21.2
	¥10,001–15,000	23	5.7
	>¥15,001	17	4.2

### Measures

To ensure sufficient content validity is satisfying, the selected measurement items were adapted mainly from prior studies but modified based on the research context of this study. More specifically, recovery justice is defined as e-shoppers’ perception of justice when e-retailers make recovery efforts (Wang et al., 2011). The scale for recovery justice was adapted from Smith et al. (1999) and Sui et al. (2013), and composed of four items: “The e-retailer handled the problem fairly in the service recovery process (RJ1),” “I was treated with courtesy and respect in the service recovery process (RJ2),” “The e-retailer had appropriate communication with me in the service recovery process (RJ3),” “The e-retailer appropriately concerned about my problem in the service recovery process (RJ4).”

Service recovery expectation is defined as e-shoppers’ expectations of what e-retailers do after service failure (McCollough et al., 2000). To correspond with recovery justice, the scale for service recovery expectation was measured with four items from McCollough et al. (2000): “Before service recovery, I had a high expectation that e-retailer would handle the problem fairly (RE1),” “Before service recovery, I had a high expectation that I would be treated with courtesy and respect (RE2),” “Before service recovery, I had a high expectation that e-retailer would communicate with me appropriately (RE3),” “Before service recovery, I had a high expectation that e-retailer would pay proper attention to my problems (RE4).”

Recovery expectation confirmation is defined as e-shoppers’ evaluation of the gap between recovery expectation and actual performance of service recovery (McCollough et al., 2000). To correspond with recovery justice, the scale for recovery expectation confirmation was assessed by four items from Fu et al. (2018): “The e-retailer handled the problem fairly, which exceeded my expectation (EC1),” “I was treated with courtesy and respect, which exceeded my expectation (EC2),” “The e-retailer had appropriate communication with me, which exceeded my expectation (EC3),” “The e-retailer appropriately concerned about my problem, which exceeded my expectation (EC4).”

Post-recovery satisfaction is defined as e-shoppers’ overall satisfaction with e-retailers after service recovery (Harris et al., 2006). The scale for post-recovery satisfaction consists of four items from Maxham and Netemeyer (2002): “I am satisfied with the procedure used to solve the problem (PS1),” “I am satisfied with the way my problem was handled with and resolved (PS2),” “I am satisfied with the overall feeling of the e-retailer attempt to make up for it (PS3),” “I am satisfied with the resources used to solve the problem (PS4).”

Customer citizenship behavior is defined as the spontaneous and voluntary actions took by e-shoppers in the network environment to reciprocate the favor of e-retailers and support the e-shops (Groth, 2005). The scale for customer citizenship behavior was measured with four items from Cheng et al. (2016): “I would like to recommend the e-shop to my peers (CB1),” “I would like to recommend the e-shop to people interested in the e-shops’ products/services (CB2),” “I would like to assist other e-shoppers in finding products (CB3),” “I would like to help other e-shoppers with their shopping (CB4).”

Respondents rated these questions on a 7-point Likert scale ranging from “1” strongly disagree to “7” strongly agree. The scales were initially developed in English and translated into Chinese by a professional translator. A reverse translation was then performed by another independent translator who is proficient in both English and Chinese to ensure that all questions are cross-linguistically comparable and express the same meaning. Furthermore, the pilot study was carried out with a sample of 30 e-shoppers to control the comprehensibility of questions in the survey.

### Analysis Procedure

The measurement model was examined by confirmatory factor analysis (CFA). Reliability (Cronbach’s alpha and composite reliability) and validity (convergent and discriminant validity) analysis were conducted to validate the scales before the main analysis phase. The structural model was tested using structural equation modeling, and the serial mediation effects were confirmed using the bootstrapping method with Mplus 7.0.

## Results

### Common Method Bias Test

A CFA was performed to test common method bias as suggested by Cheng et al. (2019). Fit indices of the hypothesized five-factor model (χ^2^/df = 2.324, CFI = 0.973, TLI = 0.968, SRMR = 0.026, RMSEA = 0.057) was much better (Δχ^2^ = 3151.174, Δdf = 10, *p* < 0.001) than that of the single-factor model (χ^2^/df = 20.724, CFI = 0.578, TLI = 0.528, SRMR = 0.142, RMSEA = 0.222), indicating that the common method bias in this study does not seem to be serious.

### Validity and Reliability of Measures

The Cronbach’s Alpha for service recovery expectation is the lowest at 0.898, followed by 0.923 for recovery justice, 0.946 for recovery expectation confirmation, 0.946 for post-recovery satisfaction, and the highest Cronbach’s Alpha for customer citizenship behavior at 0.947. All items were accepted on the basis of Cronbach’s α exceed 0.7, that is, the reliability of the instrument was satisfied (Bagozzi and Yi, 1988). As shown in Table 2, the factor loadings of all items in CFA are above 0.7. All latent constructs have obtained sufficient conditions regarding reliability and validity. According to Table 2, we can ensure that the internal consistency and convergent validity of the latent constructs have been established since the composite reliability (CR) scores are greater than 0.7 and the average variance extracted (AVE) values are greater than 0.5 (Fornell and Larcker, 1981).

**TABLE 2:** Results of measurement model.

Variables	Items	Standardized loading	AVE	CR
Service recovery expectation	RE1	0.745	0.6903	0.8988
	RE2	0.853		
	RE3	0.883		
	RE4	0.836		
Recovery justice	RJ1	0.873	0.7501	0.9231
	RJ2	0.846		
	RJ3	0.862		
	RJ4	0.883		
Recovery expectation confirmation	EC1	0.867	0.8166	0.9468
	EC2	0.936		
	EC3	0.922		
	EC4	0.888		
Post-recovery satisfaction	PS1	0.902	0.8141	0.946
	PS2	0.91		
	PS3	0.898		
	PS4	0.899		
Customer citizenship behavior	CB1	0.881	0.8174	0.9471
	CB2	0.902		
	CB3	0.913		
	CB4	0.92		

*CR, composite reliability; AVE, average variance extracted.*

As exhibited in Table 3, the square roots of the AVE of each construct are greater than the correlation coefficients between this construct and other constructs, so the discriminant validity of each construct is also supported (Fornell and Larcker, 1981). In brief, all factors in the proposed model have reached satisfactory validity.

**TABLE 3:** Correlations and square roots of AVE.

Variable	*M*	*SD*	RE	RJ	EC	PS	CB
Service recovery expectation (RE)	5.162	1.147	**0.831**				
Recovery justice (RJ)	4.768	1.129	0.511***	**0.866**			
Recovery expectation confirmation (EC)	4.190	1.169	0.198***	0.558***	**0.904**		
Post-recovery satisfaction (PS)	4.495	1.141	0.333***	0.725***	0.676***	**0.902**	
Customer citizenship behavior (CB)	4.219	1.295	0.134**	0.491***	0.535***	0.676***	**0.904**

Numbers in bold represent the square roots of AVE.

**p < 0.01, ***p < 0.001.

### Hypothesis Testing

**FIGURE 2:**
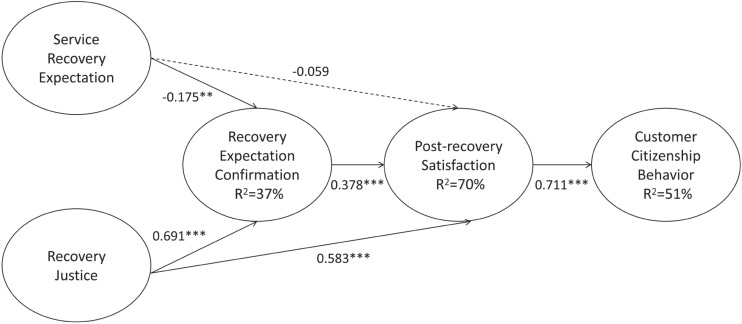
Results of the hypothesized model (Based on the most impressive service recovery experience). ^∗∗^*p* < 0.01, ^∗∗∗^*p* < 0.001. →, supported path; ⇢, unsupported path.

Mplus 7.0 was used to test the hypotheses in this study. Figure 2 exhibits the results of the hypothesized model. After accounting for the control variables, post-recovery satisfaction showed a significantly positive effect on customer citizenship behavior (β = 0.771, *p* < 0.001), and recovery expectation confirmation had a significantly positive effect on post-recovery satisfaction (β = 0.378, *p* < 0.001). Moreover, service recovery expectation had a significantly negative effect on recovery expectation confirmation (β = −0.175, *p* < 0.01). In contrast, recovery justice showed a significantly positive effect on recovery expectation confirmation (β = 0.691, *p* < 0.001) and post-recovery satisfaction (β = 0.583, *p* < 0.001), thus supporting H1, H2, H4, H5, and H6 respectively. However, there was no significant relationship between service recovery expectation and post-recovery satisfaction (β = −0.059, *p* > 0.1), thus H3 was rejected.

To verify the effects of serial mediation, a bootstrap sample of 5,000 cases with a 95% confidential interval (CI) was conducted, and the results are exhibited in Table 4. If the lower and upper levels of the 95% CI include zero, the mediation effect is insignificant. Otherwise, the mediation effect can be supported (Hayes, 2013). Table 4 presents the results of serial mediation effects. According to the results, it can be determined that the serial mediation can be confirmed. The impact of service recovery expectation on customer citizenship behavior mediated by recovery expectation confirmation and post-recovery satisfaction was negative and significant (β = –0.057) because the CI does not contain a value of 0 [–0.109, –0.019]. These research findings provide empirical support for H7. In addition, the findings showed that the impact of recovery justice on customer citizenship behavior mediated by recovery expectation confirmation and post-recovery satisfaction was significant (β = 0.200) as the CI does not contain a value of 0 [lower-level CI = 0.131; upper-level CI = 0.307]. The empirical results also provide evidence for H8.

**TABLE 4:** Bootstrapping results of serial mediation effects (Based on the most impressive service recovery experience).

				BC 95% CI	
	
Mediation effect	Estimate	Standard error	*p*	Lower	Upper
RE→EC→PS→CB	–0.057	0.023	0.011	–0.109	–0.019
RJ→EC→PS→CB	0.200	0.044	0.000	0.131	0.307

*RE, service recovery expectation; RJ, recovery justice; EC, expectation confirmation; PS, post-recovery satisfaction; CB, customer citizenship behavior.*

Although our hypothesized model showed sufficient model fit indices, we examined two alternative models to exclude alternative interpretations that seem reasonable. In the first alternative model, we assumed that service recovery expectation and recovery justice have direct effects on customer citizenship behavior instead of post-recovery satisfaction. In the second alternative model, we considered the direct impact of recovery expectation confirmation on customer citizenship behavior. Table 5 presents the results of the model fit comparisons among the hypothesized and alternative models. Compared to the two alternative models, the hypothesized model has a smaller Akaike information criterion (AIC), Bayesian information criterion (BIC), and adjusted BIC (ABIC), thus our hypothesized model has the best model fit index and offers the best way of explaining the observed patterns in our data (Wang and Wang, 2012).

**TABLE 5:** Model fit comparisons among the hypothesized and alternative models.

Models	χ2	df	*p*-value	SRMR	RMSEA	CFI	AIC	BIC	ABIC
Hypothesized model	382.892	163	<0.001	0.035	0.058	0.972	19185.158	19452.753	19240.157
Alternative Model 1	516.419	163	<0.001	0.095	0.074	0.955	19318.685	19586.281	19373.684
Alternative Model 2	520.606	164	<0.001	0.090	0.074	0.955	19320.872	19584.473	19375.050

### Supplemental Analysis

To decrease the amount of recall bias, we also collected data by asking respondents about their most recent service failure/recovery experience. In this survey, we use the same questionnaire and procedure as the first time. Six hundred questionnaires were distributed, and 414 questionnaires were collected, with a total of 373 valid questionnaires, representing a 62.2 percent response rate. In the samples, more respondents (59.8%) were male. Over two-thirds of them (61.2%) were aged from 18 to 30 years. The majority of them (81.8%) have a bachelor degree or above educational experience. 148 (39.7%) of the respondents’ average monthly income is 2,501–5,000 RMB, 103 (27.6%) of them earn 5,001–10,000 RMB, 86 (23.1%) of them earn less than 2,500 RMB, 18 (4.8%) of them earn 10,001–15,000 RMB, while 18 (4.8%) of them earn more than 15,001 RMB. The three occupations with the highest percentages are management/administrative staffs (36.5%), government officials/public servants (17.7%), and college students (11.5%). It was almost an equal split in terms of e-shopping experience: 4–6 years (26.5%), more than 6 years (23.1%), 3–4 years (22%) and 2–3 years (19.3%). Regarding e-shopping frequency, many respondents make e-shopping 4–7 times a month (37.3%).

**FIGURE 3:**
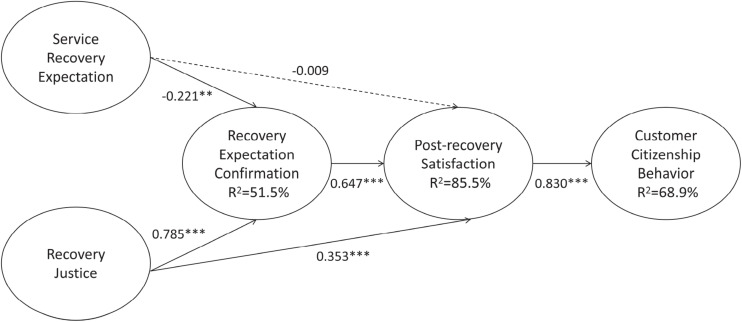
Results of the hypothesized model (Based on the most recent service recovery experience). →, supported path; ⇢, unsupported path. ^∗∗^*p* < 0.01, ^∗∗∗^*p* < 0.001.

Figure 3 exhibits the results of the hypothesized model. After accounting for the control variables, post-recovery satisfaction showed a significantly positive effect on customer citizenship behavior (β = 0.830, *p* < 0.001), and recovery expectation confirmation had a significantly positive effect on post-recovery satisfaction (β = 0.647, *p* < 0.001). Moreover, service recovery expectation had a significantly negative effect on recovery expectation confirmation (β = −0.221, *p* < 0.01). In contrast, recovery justice showed a significantly positive effect on recovery expectation confirmation (β = 0.785, *p* < 0.001) and post-recovery satisfaction (β = 0.353, *p* < 0.001), thus supporting H1, H2, H4, H5, and H6 respectively. However, there was no significant relationship between service recovery expectation and post-recovery satisfaction (β = −0.009, *p* > 0.1), thus H3 was rejected.

Table 6 presents the results of serial mediation effects. According to the results, it can be determined that the serial mediation can be confirmed. The impact of service recovery expectation on customer citizenship behavior mediated by recovery expectation confirmation and post-recovery satisfaction was negative and significant (β = –0.119), because the CI does not contain value 0 [–0.197, –0.046]. These research findings provide empirical support for H7. In addition, the findings showed that the impact of recovery justice on customer citizenship behavior mediated by recovery expectation confirmation and post-recovery satisfaction was significant (β = 0.422), as the CI does not contain a value of 0 [0.321, 0.558]. The empirical results also provide evidence for H8. In conclusion, the results of study two are in agreement with those of study one.

**TABLE 6:** Bootstrapping results of serial mediation effects (Based on the most recent service recovery experience).

				BC 95% CI	
	
Mediation effect	Estimate	Standard error	*p*	Lower	Upper
RE→EC→PS→CB	–0.119	0.038	0.002	–0.197	–0.046
RJ→EC→PS→CB	0.422	0.060	0.000	0.321	0.558

*RE, service recovery expectation; RJ, recovery justice; EC, expectation confirmation; PS, post-recovery satisfaction; CB, customer citizenship behavior.*

## Discussion

### Findings

The purpose of this study was to explore how service recovery expectation and recovery justice can promote customer citizenship behavior in the e-retailing industry. For this purpose, we used the ECT and SET to build on a research framework in which service recovery expectation and recovery justice affect customer citizenship behavior through a serial mediation of recovery expectation confirmation and post-recovery satisfaction. Our findings show that service recovery expectation negatively affects recovery expectation confirmation, while recovery justice positively affects recovery expectation confirmation. Moreover, recovery justice and recovery expectation confirmation have positive effects on post-recovery satisfaction. Post-recovery satisfaction has a significant positive effect on customer citizenship behavior. Also, recovery expectation confirmation and post-recovery satisfaction play a serial mediating role in the effect of service recovery expectation on customer citizenship behavior, as well as the relationship between recovery justice and customer citizenship behavior. Overall, the results strongly support the assertion that service recovery expectation and recovery justice can encourage customer citizenship behavior by improving recovery expectation confirmation and post-recovery satisfaction.

### Implications for Theory

The research findings of this study can contribute to the service recovery and customer citizenship behavior literature in three aspects. Although there have been significant advances in the literature focusing on customer citizenship behavior in service settings, there are few empirical studies that specifically explore customer citizenship behavior in the context of service recovery. To our best knowledge, this is the first study to focus on online service recovery and investigate how customer citizenship behavior is encouraged or impeded in the service recovery process. This study deepens our understanding of customer citizenship behavior by discovering that service recovery expectation and recovery justice affect customer citizenship behavior by inducing recovery expectation confirmation and post-recovery satisfaction.

Second, although many researchers have highlighted the role of perceived justice on customer satisfaction in the service recovery and have further analyzed the impacts of different types of justice on a customer’s satisfaction toward service recovery (Cheung and To, 2016; Gohary et al., 2016; Jung and Seock, 2017; Balaji et al., 2018; Cantor and Li, 2019), the mechanism through which recovery justice influences post-recovery satisfaction is unexplored. This study has contributed to service recovery literature by testing the mediation effect among the key variables, that is, it considers recovery expectation confirmation as a mediator in the relationship between recovery justice and post-recovery satisfaction, to confirm whether mediation analysis can support this chain of effect, thus highlighting the critical role of recovery expectation confirmation. Combined with the direct path examined with the structural equation model, the mediation effect test further elaborates the route in the service recovery process. Based on the ECT, we provide a clear process of how recovery justice enhances customers’ post-recovery satisfaction through recovery expectation confirmation. The findings open up a new avenue of service recovery research into this important but largely neglected mediating process.

Third, although service recovery studies on traditional retailing channels indicated that recovery expectation and recovery performance have significant effects on recovery expectation confirmation, which in turn affects post-recovery satisfaction (Andreaseen, 2000; McCollough et al., 2000), it is not clear whether these relationships hold true in online shopping context. Given the fact that justice is one of the essential factors for forming consumer’s view about recovery effectiveness (Maxham and Netemeyer, 2002; Del Río-Lanza et al., 2009), this study narrows this gap in recovery literature by considered recovery justice as a special type of recovery performance and confirmed the joint impacts of recovery expectation and recovery justice on recovery expectation confirmation and post-recovery satisfaction in online shopping environment.

### Implications for Practice

This study provides some useful suggestions for practitioners to perform successful service recovery, especially in the e-retailing context. The research results can be used as a guide for e-retailers to understand e-shoppers’ behavior, improve customer satisfaction and encourage customer citizenship behavior.

The significant correlation among service recovery expectation, recovery expectation confirmation, post-recovery satisfaction and customer citizenship behavior shows that recovery expectation confirmation plays a key role in service recovery management. The service recovery that fulfills consumers’ expectation is likely to lead to positive recovery expectation confirmation, thereby generating satisfied consumers, and consequently, customer citizenship behavior. Therefore, e-retailers should understand customer expectation in the recovery context and take effective measures to bridge the gap between recovery expectation and recovery justice. It can be achieved by either using data mining technology to analyze e-shopper’s preference or by asking e-shoppers how the e-retailer fix the problem so that e-retailers can fully understand e-shopper’s expectation and predict how recovery measures can satisfy the expectation of e-shopper. Due to the differences in the e-shopper’s age, occupation, education and income level, e-shopper’s expectation will vary. Therefore, when dealing with different customer expectation in online service recovery, different recovery strategies should be adopted instead of providing a standard recovery solution. Even if a customized recovery solution may be more demanding than a standard recovery solution and requires more resources to implement, it is still the most effective way to improve post-recovery satisfaction and customer citizenship behavior in the online service recovery context because e-shoppers expect more tailored recovery strategies (Mattila, 2001).

Moreover, our findings also indicate that, recovery justice has a great impact on post-recovery satisfaction and even on customer citizenship behavior. Proper service recovery can turn a perceived crisis into an opportunity to improve the effectiveness of service failure management. This finding is consistent with Weitzl and Hutzinger (2017). Therefore, a practical implication is that e-retailers need to offer sufficient financial compensation (such as refunds, replacements, coupons, and discounts), inform e-shoppers of service recovery policy, procedure as well as the recent progress of service recovery, provide an immediate response to e-shoppers’ complaints, and express a sincere apology for service failures. Furthermore, e-retailers should train front-line staffs to be knowledgeable and show courtesy, respect as well as empathy to e-shoppers in the service recovery process to ensure that they can effectively identify different service failures and respond appropriately. Besides, e-retailers should design a well-established recovery system to respond to service failures promptly. In this way, e-retailers can raise e-shoppers’ satisfaction toward the recovery outcomes and process and ultimately affect customer citizenship behavior. Given that it costs more to acquire new customers than to retain existing ones, generosity in service recovery can reward e-retailers in the long term.

We also found that e-shoppers’ post-recovery satisfaction has a significant impact on customer citizenship behavior. This conclusion supports previous findings which confirmed the remarkable effect of satisfaction on behavioral intention (Jones and Suh, 2000; Zboja and Voorhees, 2006). Therefore, e-retailers should make sure that e-shopper’s post-recovery satisfaction is achieved. They should strive to keep track of e-shoppers so that e-retailers can get prompt and actionable feedback, which may lead to e-shoppers’ post-recovery satisfaction. If e-shoppers are satisfied with service recovery, they are more inclined to implement customer citizenship behavior.

### Limitations and Future Research

Although this study has made some interesting findings, it also has some limitations. Firstly, considering that individual cognition and affection can vary with time, recovery expectation and recovery justice should be examined longitudinally. Therefore, one limitation of this study is that we use a cross-sectional survey method, which can only display a snapshot of variables at a certain moment but cannot accurately reveal the dynamic connections. Since this exists in all cross-sectional surveys (Gallivan et al., 2005), longitudinal research is needed in the future. Another limitation of this work is that all data is self-reported by the surveyed e-shoppers. Since the respondents may conceal their true thoughts, future studies should use other data collection methods to obtain more abundant data and make further interpretation of data analysis. Finally, there are other factors related to post-recovery satisfaction that may also play a role. Future research should include other variables, such as customer relationship (Hess et al., 2003) and recovery attributes (Webster and Sundaram, 1998).

## Data Availability Statement

The raw data supporting the conclusions of this article will be made available by the authors, without undue reservation.

## Author Contributions

TZ contributed to the conceptualization, funding acquisition, and investigation. BL contributed to the investigation. MS contributed to the formal analysis. JW contributed to the conceptualization and methodology. All authors contributed to the article and approved the submitted version.

## Conflict of Interest

The authors declare that the research was conducted in the absence of any commercial or financial relationships that could be construed as a potential conflict of interest.

## Footnotes

1 www.wenjuan.com
